# Upper Limb Nerve Transfer Surgery in Patients With Tetraplegia

**DOI:** 10.1001/jamanetworkopen.2022.43890

**Published:** 2022-11-28

**Authors:** Saad Javeed, Christopher F. Dibble, Jacob K. Greenberg, Justin K. Zhang, Jawad M. Khalifeh, Yikyung Park, Thomas J. Wilson, Eric L. Zager, Amir H. Faraji, Mark A. Mahan, Lynda J. Yang, Rajiv Midha, Neringa Juknis, Wilson Z. Ray

**Affiliations:** 1 Department of Neurological Surgery, Washington University, St Louis, Missouri; 2 Department of Neurological Surgery, Johns Hopkins University, Baltimore, Maryland; 3 Division of Public Health Sciences, Department of Surgery, Washington University School of Medicine, St Louis, Missouri; 4 Department of Neurosurgery, Stanford University, Stanford, California; 5 Department of Neurosurgery, Hospital of the University of Pennsylvania, Philadelphia; 6 Department of Neurological Surgery, Houston Methodist Hospital, Houston, Texas; 7 Department of Neurosurgery, Clinical Neurosciences Center, The University of Utah, Salt Lake City; 8 Department of Neurological Surgery, University of Michigan School of Medicine, Ann Arbor; 9 Department of Clinical Neurosciences, University of Calgary, Calgary, Alberta, Canada; 10 Physical Medicine and Rehabilitation, Washington University, St Louis, Missouri

## Abstract

**Importance:**

Cervical spinal cord injury (SCI) causes devastating loss of upper extremity function and independence. Nerve transfers are a promising approach to reanimate upper limbs; however, there remains a paucity of high-quality evidence supporting a clinical benefit for patients with tetraplegia.

**Objective:**

To evaluate the clinical utility of nerve transfers for reanimation of upper limb function in tetraplegia.

**Design, Setting, and Participants:**

In this prospective case series, adults with cervical SCI and upper extremity paralysis whose recovery plateaued were enrolled between September 1, 2015, and January 31, 2019. Data analysis was performed from August 2021 to February 2022.

**Interventions:**

Nerve transfers to reanimate upper extremity motor function with target reinnervation of elbow extension and hand grasp, pinch, and/or release.

**Main Outcomes and Measures:**

The primary outcome was motor strength measured by Medical Research Council (MRC) grades 0 to 5. Secondary outcomes included Sollerman Hand Function Test (SHFT); Michigan Hand Outcome Questionnaire (MHQ); Disabilities of Arm, Shoulder, and Hand (DASH); and 36-Item Short Form Health Survey (SF-36) physical component summary (PCS) and mental component summary (MCS) scores. Outcomes were assessed up to 48 months postoperatively.

**Results:**

Twenty-two patients with tetraplegia (median age, 36 years [range, 18-76 years]; 21 male [95%]) underwent 60 nerve transfers on 35 upper limbs at a median time of 21 months (range, 6-142 months) after SCI. At final follow-up, upper limb motor strength improved significantly: median MRC grades were 3 (IQR, 2.5-4; *P* = .01) for triceps, with 70% of upper limbs gaining an MRC grade of 3 or higher for elbow extension; 4 (IQR, 2-4; *P* < .001) for finger extensors, with 79% of hands gaining an MRC grade of 3 or higher for finger extension; and 2 (IQR, 1-3; *P* < .001) for finger flexors, with 52% of hands gaining an MRC grade of 3 or higher for finger flexion. The secondary outcomes of SHFT, MHQ, DASH, and SF36-PCS scores improved beyond the established minimal clinically important difference. Both early (<12 months) and delayed (≥12 months) nerve transfers after SCI achieved comparable motor outcomes. Continual improvement in motor strength was observed in the finger flexors and extensors across the entire duration of follow-up.

**Conclusions and Relevance:**

In this prospective case series, nerve transfer surgery was associated with improvement of upper limb motor strength and functional independence in patients with tetraplegia. Nerve transfer is a promising intervention feasible in both subacute and chronic SCI.

## Key Points

**Question:**

Does nerve transfer surgery improve upper limb motor strength and functional independence after cervical spinal cord injury?

**Findings:**

In this prospective case series of 22 patients with tetraplegia, nerve transfer surgery after tetraplegia was associated with improved upper limb motor strength and increased functional independence. Nerve transfers in subacute and chronic spinal cord injury resulted in comparable outcomes, and continual motor recovery was observed after surgery.

**Meaning:**

Early and late nerve transfer surgery in patients with tetraplegia is feasible and can successfully reanimate upper limb function.

## Teaser

This case series examines whether nerve transfers are associated with restored upper extremity function in patients with tetraplegia.

## Introduction

Spinal cord injury (SCI) leads to chronic impairment and disability.^1^ The incidence of traumatic SCI is 54 cases per million, with 17 900 new injuries per annum.^2^ Of the 296 000 patients living with this condition, 50% have cervical SCI, resulting in varying degrees of motor and sensory loss in the upper and lower extremities that causes tetraplegia.^2^ Patients with tetraplegia rate restoration of hand function among their highest priorities.^3^ Even partial gains in arm or hand control can have a profound effect on functional independence and quality of life.^4,5^ Restoration of elbow extension and hand grasp, pinch, and release can maximize independence in mobility, feeding, grooming, and self-catheterization.^3,6^ In mid- to low-cervical SCI, one option for restoring these functions is tendon transfers. These transfers redirect remaining volitional motor function onto select paralyzed muscles affected by the SCI, permitting a 1-to-1 donor-to-recipient exchange of motor function.^7,8^

An alternative option for restoring function is nerve transfer, which aims to reanimate paralyzed muscles of the upper extremity. The rationale underlying nerve transfer in tetraplegia is redirecting healthy proximal nerve axons that originate above the zone of injury onto paralyzed muscle-nerve units caudal to the zone of injury, effectively bypassing the injured segment of the spinal cord. Healthy axons that are under volitional control regenerate from the donor nerves to restore control of previously paralyzed muscles below the SCI.^9^ Unlike tendon transfers, nerve transfers preserve native musculoskeletal mechanical advantages and do not require prolonged limb immobilization. Moreover, nerve transfers leverage regenerating nerves’ potential for collateral axonal sprouting, such that 1 motor axon can reinnervate more than 5 recipient motor axons to control multiple muscles.^10,11,12^ With this 1:5 axonal exchange, a single donor nerve can reinnervate a large recipient territory, providing excellent control and dexterity, with minimal loss of donor function.^13^

Although there is an increasing acceptance and evolution of nerve transfers in SCI, the indications, optimal timing, patient selection, and long-term clinical outcomes remain poorly understood. Addressing this shortcoming, we report the results of, to our knowledge, the largest prospective case series of nerve transfers to restore upper extremity function in patients with tetraplegia.

## Methods

### Study Design and Participants

Between September 1, 2015, and January 31, 2019, a total of 153 patients were screened for eligibility, and 22 patients with traumatic cervical SCI were eligible and recruited (eFigure 1 in the Supplement). The preliminary results of this study were previously reported.^14^ We enrolled adults with cervical SCI with American Spinal Injury Association Impairment (ASIA) grades A to C in whom recovery plateaued for at least 6 months. Because patients’ race and socioeconomic background may impact their access to tertiary health care and SCI outcomes,^15^ we attempted to enroll all patients regardless of their race or ethnicity, which is imprtant for the clinical implementation of this intervention. Participants self-reported their race at the time of enrollment. Detailed eligibility criteria are given in eTable 1 in the Supplement. Patients in International Classification for Surgery of the Hand in Tetraplegia (ICSHT)^16^ groups 0 to 4 were eligible for nerve transfers (eTable 2 in the Supplement). Preoperative nerve conduction studies and electromyography were performed to evaluate nerve and muscle integrity associated with the SCI (eTable 3 in the Supplement). Functional electrical stimulation verified lower motor neuron firing in the target recipient muscles. Patients were excluded if they had conditions that would limit recovery after nerve transfers (eg, joint contractures). A timeline of assessments and interventions is available in eTable 4 in the Supplement. Written informed consent was obtained from all study participants. This prospective case series has been reported in line with the PROCESS guidelines.^17^ Ethics approval was obtained from the institutional review board at Washington University, and the study was registered in ClinicalTrials.gov.^18^

### Interventions

**Figure 1.**
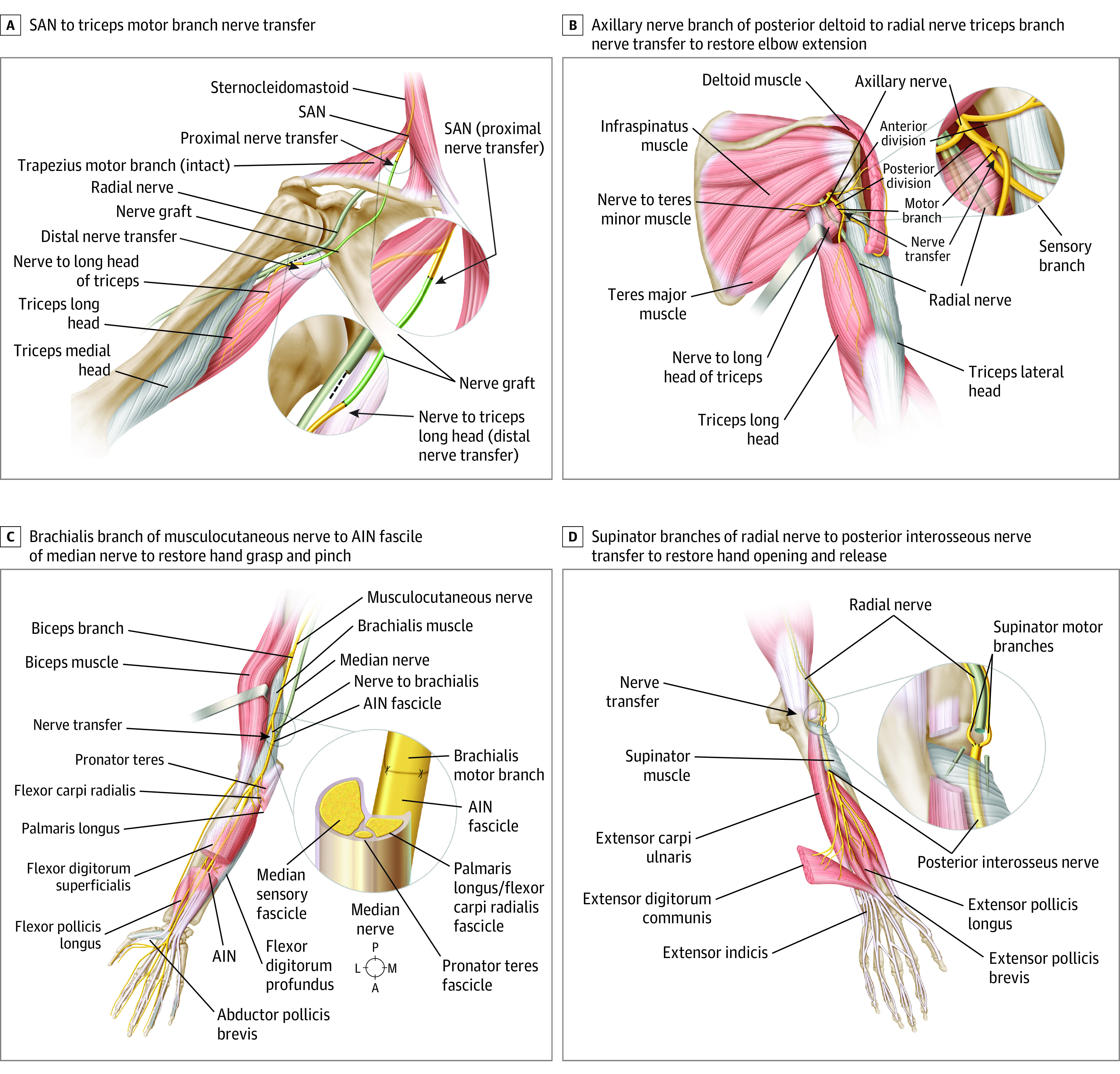
Nerve Transfers Only muscles innervated by recipient nerves have restoration of activity (gray represents atrophic muscles not innervated). In high tetraplegia (International Classification for Surgery of the Hand in Tetraplegia group 0), no upper extremity donor nerves are available. The spinal accessory nerve (SAN) is transferred to the triceps motor branch using an approximately 20-cm interpositional nerve graft to restore elbow extension. The brachialis motor branch is transferred to the isolated anterior interosseus nerve (AIN) fascicle in the proximal median nerve. A indicates anterior; L, lateral; M, medial; P, posterior.

All patients underwent single, double, or triple nerve transfers to restore upper extremity function in one or both limbs. Nerve transfers were chosen based on the (1) level of injury, (2) residual motor function per ICSHT grouping, and (3) electrodiagnostic patterns of motor neuron injury. Donor nerves were selected if they had clinically functional motor strength, defined as a Medical Research Council (MRC) grade of 4 to 5. Nerve transfer pairings to restore target functions included (1) posterior deltoid motor branch of the axillary nerve to triceps branch of the radial nerve to reanimate elbow extension, (2) supinator branch of the radial nerve to posterior interosseus nerve (PIN) to reanimate hand opening and finger and wrist extension, (3) brachialis branch of the musculocutaneous nerve to anterior interosseus nerve (AIN) fascicle of the median nerve to reanimate pinch and finger flexion, and (4) for patients with high cervical SCI (C4 level and above, ICSHT group 0), use of the spinal accessory nerve (SAN) to reinnervate the target recipient muscles (Figure 1).

During surgery, intraoperative electrical stimulation (Checkpoint Surgical) was used to ensure normal lower motor neuron connectivity in donor and recipient nerves. Nerves were coapted in a tension-free manner with 8-0 nylon suture reinforced by fibrin glue (Tisseel, Baxter). After nerve transfer, the upper extremity was immobilized temporarily for 1 week in a sling, limiting reaching, pushing, or pulling over the elbow or shoulder to protect the coaptation. After immobilization, occupational hand therapy was initiated, with each session lasting for 1 hour daily for 6 weeks followed by once weekly until 24 to 36 months after nerve transfer. Recipient muscles were worked in a passive range of motion, then donor co-contraction and repetition exercises began to enhance the recruitment of donor axons.^19^ Once muscles demonstrated signs of reinnervation (eg, palpable contraction), incremental resistance training was started with task-oriented activities to enhance cortical relearning and functional use.^12,19^

### Outcomes

Outcomes were assessed preoperatively and 2, 6, 12, 18, 24, 36, and 48 months postoperatively. The primary outcome was motor strength in MRC grades (eTable 5 in the Supplement). The secondary outcomes were scores on the Sollerman Hand Function Test (SHFT) (range, 0-80; higher scores indicate increased function); the Michigan Hand Outcome Questionnaire (MHQ) (range, 0-100; higher scores indicate better function); the Disabilities of Arm, Shoulder, and Hand (DASH) (range, 0-100; lower scores indicate improved impairment); and the 36-Item Short Form Health Survey (SF-36), version 2 physical component summary (PCS) and mental component summary (MCS) (range, 0-100; higher scores indicate better health). The SHFT was administered by an occupational hand therapist only in participants who underwent nerve transfers to reanimate hand function. The SHFT is a tetraplegia-specific measure to evaluate the ability of the hand to perform various activities of daily living (ADLs).^20,21^ The MHQ has the capability to evaluate individual upper limbs separately and has well-defined clinical benchmarks in patients with upper extremity disorders.^22,23^ Because several patients underwent bilateral nerve transfers, the MHQ allowed evaluation of each upper limb separately. DASH has been widely used to evaluate upper limb impairment in patients with stroke and cervical spondylotic myelopathy.^24,25^

### Statistical Analysis

Data analysis was performed from August 2021 to February 2022. Categorical variables are presented as numbers (percentages), and continuous data are reported as median (IQR) or mean (SD) as appropriate. Improvement from baseline to final follow-up outcomes was analyzed using Wilcoxon signed rank tests. Latest available follow-up was considered as the final time point. Missing follow-up data between initial and final time points were imputed using a last observation carried forward approach. Time to reinnervation and time to motor grade 3 or higher were analyzed using survival analysis and log-rank tests. Improvement in motor strength over time at 12-, 24-, and 48-month follow-up was analyzed using the Friedman test, a nonparametric test examining trends in improvement across repeated assessments. Pairwise comparisons between measures at 12 to 24, 12 to 48, and 24 to 48 months were analyzed using Wilcoxon rank sum tests with Bonferroni correction. Subgroup analyses assessed the influence of time delay after SCI and degree of preserved upper limb function (ICSHT grouping) on motor outcomes.^16,26^ The threshold for significance was set at a 2-sided α < .05. Spearman rank-order correlation was used to assess the association of primary and secondary outcome measures with Bonferroni correction to adjust the α level for multiple correlations. The correlation coefficients, Spearman ρ (r_s_), were considered weak if r_s_ < 0.3, moderate if r_s_ = 0.3 to 0.5, and strong if r_s_ ≥ 0.5.^27^ Established minimal clinically important difference (MCID) metrics of secondary outcome measures were used (MHQ, 9.3 points; MHQ-ADLs, 14.7 points^28^; DASH, 10.83 points^29^; and SF-36 PCS and MCS, 4 points^30^). Because no validated metric for SHFT exists, the MCID of SHFT was defined by a distribution-based method (SEM = SD × sqrt[1 − R]), where SD is the standard deviation of the baseline SHFT score and R is the test-retest reliability of SHFT, which is established to be 0.9.^20^ The MCID of SHFT was defined as 4.9 points. All data analyses were performed in R, version 4.2.1 (R Foundation for Statistical Computing).

## Results

### Demographic Data

Twenty-two patients with tetraplegia (median age, 36 years [range, 18-76 years]; 21 male [95%] and 1 female [5%]; 19 White [86%] and 3 African American [14%]) were included in the study. Demographic characteristics are given in Table 1. The most common cause of SCI was motor vehicle collisions (11 patients [50%]), with the preoperative neurologic level of injury ranging from C2 to C7. Overall, 19 patients (86%) had motor complete SCI (ASIA A-B).

**Table 1.** Demographic Characteristics

Characteristic	No. (%) of patients (N = 22)
Age, median (range), y	35 (18-76)
Sex	
Male	21 (95)
Female	1 (5)
Race	
African American	3 (14)
White	19 (86)
Time after SCI, median (range), mo	21 (6-142)^a^
Mechanism of injury	
Motor vehicle collision	11 (50)
Sports injury	7 (32)
Assault	2 (9)
Fall	1 (4.5)
Iatrogenic	1 (4.5)
ASIA grade	
A	10 (45)
B	9 (41)
C	3 (14)^b^
Neurologic level of SCI	
C1-C4	14 (64)
C5-C8	8 (36)
Preoperative hand dominance	
Right	21 (95)
Left	1 (5)
No. of upper limbs	35

Abbreviations: ASIA, American Spinal Injury Association Impairment Scale; SCI, spinal cord injury.

^a^ One patient underwent nerve transfer at 142 months of SCI (deviation from <60 months in criteria).

^b^ One patient had SCI ASIA grade D central cord syndrome (deviation from ASIA grades A-C inclusion criteria).

Sixty nerve transfers were performed on 35 upper limbs at a median time of 21 months (range, 6-142 months) after initial SCI. Among these nerve transfers, 26 (43%) were brachialis motor branch to AIN, 20 (33%) were supinator motor branch to PIN, 4 (7%) were posterior axillary nerve (deltoid motor branch) to triceps motor branch, and 1 (2%) was flexor carpi radialis/flexor digitorum superficialis motor fascicle to biceps motor branch. In 4 patients with high cervical SCI (C4 and above, ICSHT group 0), 7 upper limbs were reanimated using cranial donor nerves (SAN or platysma motor branch), comprising 9 nerve transfers (15%). Thirteen patients (59%) underwent staged bilateral nerve transfers (Table 2).

**Table 2.** Patient Characteristics, Spinal Cord Injury Classification, Surgical Procedures, and Final Postoperative Outcomes in 22 Patients With Tetraplegia

Patient No./age group, y/sex	NLI	ASIA grade	TIS, mo	Follow-up, mo	Limb	Nerve transfers	Motor outcomes
**ICSHT group 0**							
1/10-19/M	C4	B	52	26	R	SAN to triceps branch of radial nerve (sural graft)	Triceps: 3
			52	25	L	SAN to middle trunk of brachial plexus-triceps motor component	Triceps: 3
2/20-29/M	C2	A	6	37	R	Platysma to triceps (sural graft), SAN to biceps (MABC graft)	Triceps: 0, biceps: 1
			6	37	L	Platysma to triceps (sural graft), SAN to biceps (MABC graft)	Triceps: 0, biceps: 0
3/30-39/M	C3	A	11	25	R	SAN to AIN, FDS, FCR (sural graft)	FDP: 0, FPL: 0, FCR: 0
4/30-39/M	C4	A	139	17	R	SAN to triceps branch of radial nerve (sural graft)	Triceps: 4
			142^a^	14	L	SAN to triceps branch of radial nerve	Triceps: 3
**ICSHT group 1**							
5/50-59/M	C5	A	8	37	R	Brachialis to AIN	FDP: 1, FPL: 0, FCR: 1
			9	37	L	Brachialis to AIN	FDP: 2, FPL: 0, FCR: 1
6/30-39/M^b^	C4	A	61	6	L	Brachialis to AIN, supinator to PIN	NA
7/20-29/M	C4	A	39	25	L	Brachialis to AIN, supinator to PIN	FDP: 1, FPL: 2, FCR: 0, EDC: 1, EPL/B: 3
			46	19	L	Axillary to triceps	Triceps: 2
8/30-39/M	C4	A	21	42	R	Brachialis to AIN, supinator to PIN	FDP: 2, FPL: 0, FCR: 0, EDC: 0, EPL/B: 0
			26	37	L	Brachialis to AIN	FDP: 2, FPL: 1, FCR: 1
9/30-39/M	C4	B	21	33	L	Brachialis to AIN, supinator to PIN	FDP: 3, FPL: 1, FCR: 0, EDC: 5, EPL/B: 4
**ICSHT group 2**							
10/20-29/M	C4	B	44	23	R	Brachialis to AIN, supinator to PIN	FDP: 1, FPL: 1, FCR: 0, EDC: 1, EPL/B: 1
			44	23	L	Brachialis to AIN, supinator to PIN	FDP: 2, FPL: 0, FCR: 0, EDC: 3, EPL/B: 2
**ICSHT group 3**							
11/20-29/M	C4	C	9	26	R	Brachialis to AIN, supinator to PIN, axillary to triceps	FDP: 2, FPL: 3, FCR: 2, EDC: 3, EPL/B: 3, triceps: 4
12/20-29/M	C6	B	25	42	R	Brachialis to AIN, supinator to PIN	FDP: 3, FPL: 3, FCR: 2, EDC: 3, EPL/B: 3,
			15	52	L	Brachialis to AIN, supinator to PIN	FDP: 4, FPL: 3, FCR: 3, EDC: 2, EPL/B: 2
13/10-19/M	C6	B	24	51	R	Brachialis to AIN, supinator to PIN, axillary to triceps^c^	FDP: 1, FPL: 1, FCR: 0, EDC: 2, EPL/B: 4, triceps: NA
			24	51	L	Brachialis to AIN	FDP: 1, FPL: 1, FCR: 0
14/40-49/M	C5	A	15	47	L	Brachialis to AIN, supinator to PIN	FDP: 3, FPL: 2, FCR: 1, EDC: 4, EPL/B: 4
15/40-49/M	C4	B	21	49	R	Brachialis to AIN, supinator to PIN	FDP: 3, FPL: 3, FCR: 2, EDC: 4, EPL/B: 4
16/20-29/M	C4	A	13	39	R	Brachialis to AIN, supinator to PIN	FDP: 2, FPL: 2, FCR: 0, EDC: 3, EPL/B: 3
			10	42	L	Brachialis to AIN, supinator to PIN	FDP: 3, FPL: 3, FCR: 0, EDC: 4, EPL/B: 3
17/40-49/M	C4	B	17	21	R	Brachialis to AIN, supinator to PIN, axillary to triceps	FDP: 2, FPL: 1, FCR: 1, EDC: 3, EPL/B: 3, triceps: 3
			22	17	L	Brachialis to AIN, supinator to PIN, axillary to triceps	FDP: 2, FPL: 1, FCR: 1, EDC: 1, EPL/B: 3, triceps: 3
18/20-29/M	C7	A	7	29	R	Brachialis to AIN, supinator to PIN	FDP: 4, FPL: 3, FCR: 4, EDC: 1, EPL/B: 1
			7	29	L	Brachialis to AIN	FDP: 3, FPL: 3, FCR: 4
**ICSHT group 4**							
19/40-49/F	C6	B	13	29	R	Brachialis to AIN	FDP: 1, FPL: 1, FCR: 5
20/10-19/M	C7	C	49	37	R	Brachialis to AIN, supinator to PIN	FDP: 2, FPL: 2, FCR: 4, EDC: 3, EPL/B: 3
			38	48	L	Brachialis to AIN, supinator to PIN	FDP: 3, FPL: 3, FCR: 4, EDC: 3, EPL/B: 4
21/30-39/M	C5	B	26	49	R	Brachialis to AIN, supinator to PIN	FDP: 4, FPL: 4, FCR: 2, EDC: 4, EPL/B: 4
**Other**							
22/70-79/M	C2	D^a^	18	51	R	FDS, FCR to biceps branch of musculocutaneous nerve	Biceps: 3

Abbreviations: ASIA, American Spinal Injury Association Impairment scale; AIN, anterior interosseus nerve; EDC, extensor digitorum communis; EPL/B, extensor pollicis longus/brevis; F, female; FCR, flexor carpi radialis; FDP, flexor digitorum profundus; FDS, flexor digitorum superficialis; FPL, flexor pollicis longus; ICSHT, International Classification for Surgery of the Hand in Tetraplegia; M, male; MABC, medial antebrachial cutaneous nerve; NA, not availabe; NLI, neurologic level of injury; PIN, posterior interosseus nerve; SAN, spinal accessory nerve; TIS, time interval from spinal cord injury to surgery.

^a^ Deviation from initial eligibility criteria in participant 4 (nerve transfers were performed at 139 and 142 months) and participant 22 (ASIA grade D spinal cord injury central cord syndrome).

^b^ Participant 6 was deceased at 6 months’ follow-up.

^c^ Nerve transfer was deferred because of negative intraoperative electrical stimulation.

### Primary Outcome

Motor outcomes were assessed for a median follow-up of 37 months (IQR, 25-42 months). Preoperatively, all recipient nerve muscles had absent motor function, and all donor nerve muscles had a motor grade of 4 to 5. Postoperatively, no meaningful or lasting donor site deficits in terms of motor downgrade were reported (eTables 6 and 7 in the Supplement). Among our cohort, 2 participants (9%) and 3 upper limbs (9%) had no more than 24 months of follow-up measures available (Table 2).

**Figure 2.**
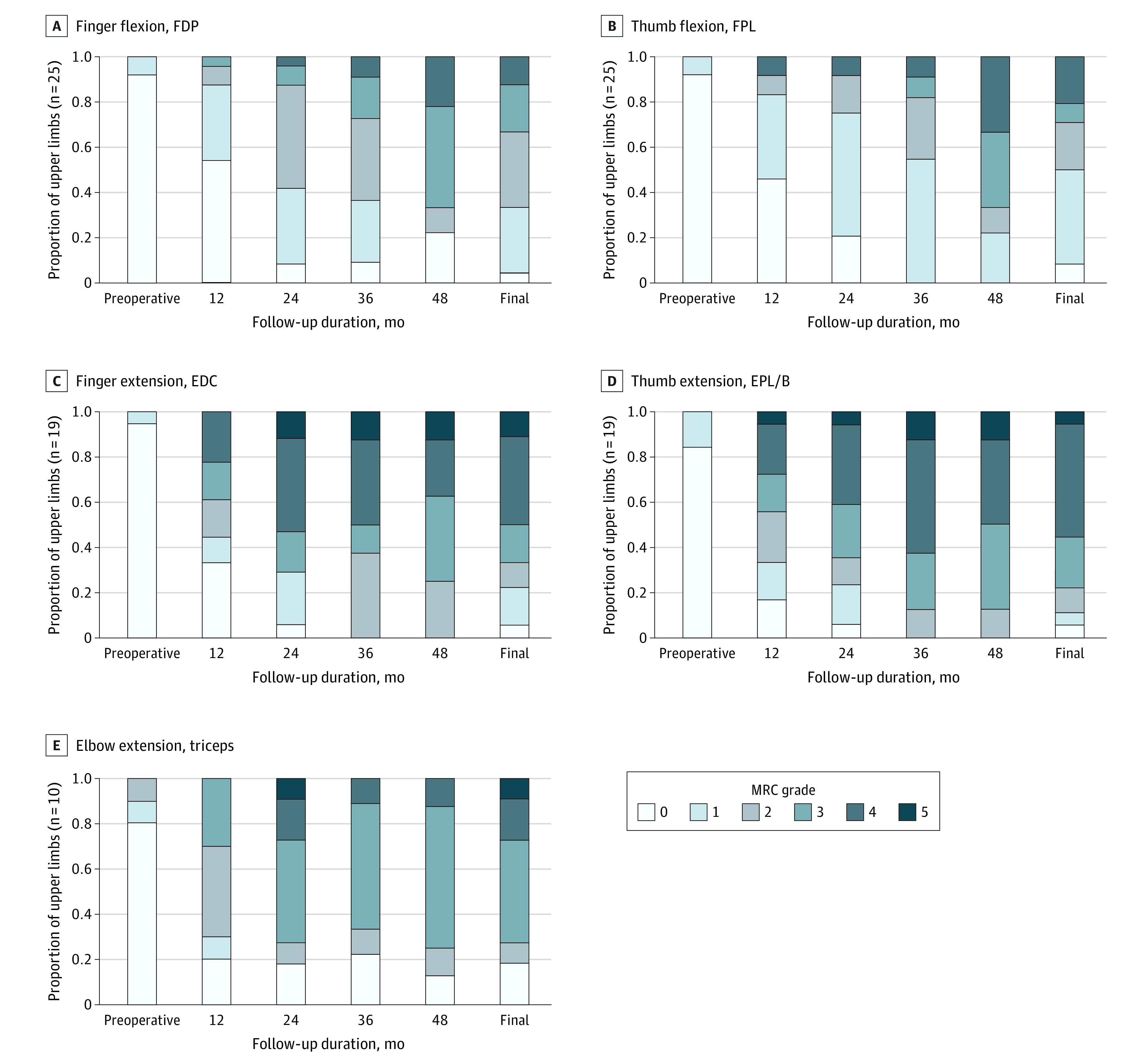
Incremental Change in Motor Strengths of Flexor and Extensor Muscles Recorded at Baseline and at Each Follow-up Evaluation Missing data between initial visit and final follow-up were imputed using the last observation carried forward approach between before and after time points. Gradient bars demonstrate Medical Research Council (MRC) grade of 0 to 5. Paired Wilcoxon signed rank tests were used to analyze preoperative and final postoperative MRC scores. Flexor digitorum profundus (FDP) in brachialis to anterior interosseus nerve and extensor digitorum communis (EDC) in supinator to posterior interosseus nerve and triceps in axillary to triceps nerve transfer were used as surrogates to demonstrate evidence of reinnervation and gaining meaningful function. Reinnervation was defined as gaining palpable contraction of an MRC grade of 1, and antigravity was defined as gaining an MRC grade of 3 or higher at subsequent follow-up evaluations. Log-rank tests were used to analyze the significance of difference in gaining reinnervation and meaningful function. Final indicates the last recorded motor strength for each patient; EPL/B, exensor pollicis longus/brevis; and FPL, flexor pollicis longus.

Statistically significant differences were found in motor strength of recipient nerve muscles between baseline and final follow-up (Figure 2, Table 2). After elbow extension reanimation, 7 upper limbs (70%) achieved a motor grade of 3 or higher, and the median postoperative triceps MRC grade was 3 (IQR, 2.5-4; *P* = .01) (corresponding to SAN and axillary nerve to triceps in eTable 7 in the Supplement). All triceps reinnervated using a SAN donor nerve achieved a motor grade of 3 or higher (Table 2). Functional elbow extension recovery in participants is shown in Video 1. After 1 flexor digitorum superficialis/flexor carpi radialis fascicle to biceps nerve transfer, the patient achieved a motor grade of 3 for biceps function (Table 2).

**Video 1.** Nerve Transfer Surgery to Restore Function in Patients With Spinal Cord Injury: Elbow Function This video shows the recovery of elbow extension (ie, triceps) function after nerve transfer surgery in patients with cervical spinal cord injury, also known as tetraplegia. Before surgery, both patients in this video had no upper limb function. In the first patient, the nerve supplying the posterior deltoid muscle was used as donor nerve and transferred to the nerve supplying the triceps muscle. In the second patient, the nerve supplying the trapezius muscle was used as donor nerve and transferred to the nerve supplying the triceps muscle. After nerve transfer surgery, both patients gained full functional strength in the triceps muscles.

After reanimation of finger and thumb extension, 15 of 19 hands (79%) achieved a motor grade of 3 or higher for function (median postoperative finger extension [extensor digitorum communis (EDC)] MRC grade, 4; IQR, 2-4; *P* < .001; median thumb extension [extensor pollicis longus/brevis (EPL/B)] MRC grade, 4; IQR, 3-4; *P* < .001) (eTable 7 in the Supplement). A motor grade of 3 or higher for hand grasp and finger and thumb flexion was achieved in 13 of 25 hands (52%) after nerve transfer (median postoperative finger flexion [flexor digitorum profundus (FDP)] and thumb flexion [flexor pollicis longus (FPL)] MRC grade, 2; IQR, 1-3; *P* < .001 for both) (eTable 7 in the Supplement). Results of participants with double nerve transfers to reanimate grasp, pinch, and hand opening are shown in Video 2 (eFigure 2 in the Supplement).

**Video 2.** Nerve Transfer Surgery to Restore Function in Patients With Spinal Cord Injury: Hand Function This video shows the recovery of hand function after nerve transfer surgery in patients with cervical spinal cord injury, also known as tetraplegia. Patents shown in this video were not able to open and close their hands. The nerve supplying the supinator muscle was used as donor nerve and transferred to the posterior interosseous nerve, which controls muscles for hand opening. The nerve supplying the brachialis muscle was used as donor nerve and transferred to the anterior interosseous nerve, which controls muscles for hand closing. After nerve transfer surgery, patients were able to open and close their hands independently.

The trajectory of reinnervation and motor recovery in the recipient muscles is shown in Figure 2. After nerve transfers, the triceps had the shortest time to reinnervation, with a median of 7 months (95% CI, 3-11 months), followed by EDC innervated by PIN at 12 months (95% CI, 7.4-16.6 months) and FDP innervated by AIN at 18 months (95% CI, 15-21 months); however, the differences in motor reinnervation periods between nerve transfers were nonsignificant (log-rank test *P* = .20) (Figure 2). On analyzing the time to motor grade 3 (antigravity function), triceps gained earlier motor function with a median of 17 months (95% CI, 16-18 months), followed by EDC at 18 months (95% CI, 16-19 months) and FDP at 48 months (95% CI, 46-49 months) postoperatively; the recovery kinetics were significantly different (log-rank test *P* = .004) (Figure 2).

The improvements in motor strength in the recipients across multiple assessments are detailed in eTable 8 and eFigure 3 in the Supplement. A statistically significant improvement was seen in finger and thumb flexor strength (FDP: median MRC, 1 [IQR, 0-1] at 12-month follow-up, 2 [1-2] at 24-month follow-up, and 3 [IQR, 2-3] at 48-month follow-up; Friedman test *P* < .001; FPL: median MRC, 1 [IQR, 0-1] at 12-month follow-up, 21 [IQR, 1-2] at 24-month follow-up, and 3 [IQR, 1-4] at 48-month follow-up; Friedman test *P* < .001) (eTable 8 and eFigure 3 in the Supplement). Similarly, finger and thumb extensor strength improved (EDC: median MRC, 2 [IQR, 0-3] at 12-month follow-up, 3.5 [IQR, 2-4] at 24-month follow-up, and 4 [IQR, 3-4] at 48-month follow-up; *P* = .003; EPL/B: median MRC, 2 [IQR, 1-3] at 12-month follow-up, 3.5 [IQR, 3-4] at 24-month follow-up, and 4 [IQR, 3-4] at 48-month follow-up; *P* = .004). Triceps motor strength plateaued at 12 months with nonsignificant changes afterward (triceps: median MRC, 3 [IQR, 1-3] at 12-month follow-up, 3 [IQR, 2-4] at 24-month follow-up, and 3 [IQR, 0-4] at 48-month follow-up; *P* = .14).

On subgroup analysis, early nerve transfers (performed ≤12 months after SCI) had comparable motor outcomes to delayed nerve transfers (≥12 months after SCI) (eTable 9 in the Supplement). However, nerve transfers performed in upper limbs with lower-level injury (ICSHT groups 3-4) had favorable motor outcomes compared with those performed on high-level injuries (ICSHT groups 0-2) (median MRC, 4 (3-4) for lower-level injury vs 2 [IQR, 1-3] for high-level injuries; Mann-Whitney test *P* < .001) (eTable 9 in the Supplement).

### Secondary Outcomes

**Figure 3.**
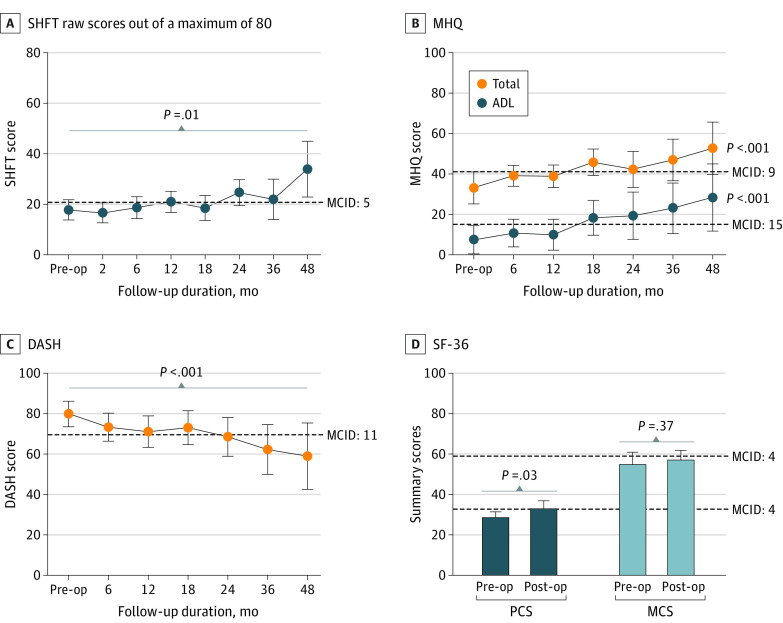
Gain in Postoperative Hand Function and Patient-Reported Outcome Measures The Sollerman Hand Function Test (SHFT) scores are raw scores of a maximum of 80. To avoid confounding with hand functional scores, triceps nerve transfers were excluded from SHFT and only nerve transfers to reanimate hand function were included. The Michigan Hand Outcome Questionnaire (MHQ) total cumulative scores and activities of daily living (ADLs) component were normalized to 100. The Disabilities of Arm, Shoulder, and Hand (DASH) scores were normalized to 100. The 36-Item Short Form Health Survey (SF-36) physical component summary (PCS) and mental component summary (MCS) scores were normalized to 100. Preoperative (pre-op) and final postoperative (post-op) values were analyzed via a paired Wilcoxon signed rank test. Error bars represent 95% CIs. Dotted lines represent the minimal clinically important difference (MCID) threshold from mean baseline scores.

Between baseline and final follow-up, the SHFT and MHQ scores improved significantly. Median improvement was 5 points (95% CI, 2-9.5 points; *P* = .01) in the SHFT and 15 points (95% CI, 8-22 points; *P* < .001) in the MHQ, both greater than the MCID (Figure 3; eTable 10 in the Supplement). In addition, significant improvements were observed on the ADLs, work performance, and satisfaction with hand function scores on the MHQ; all metrics improved greater than the MCID (eTable 10 in the Supplement). Improvement in the SHFT scores had a strong positive correlation with total MHQ scores (r_s_ = 0.66), hand function (r_s_ = 0.69), ADLs (r_s_ = 0.77), and satisfaction with hand function (r_s_ = 0.59) (eFigure 4 in the Supplement).

At final follow up, the DASH score improved by a median of 13.3 points (95% CI, 8-21.7 points; *P* < .001), and the SF-36 PCS score improved by a median of 5.6 points (95% CI, 1.3-8.2 points; *P* = .03). However, no significant improvement was found in the SF-36 MCS score (median, 2 points; 95% CI, −3.3 to 6.6 points; *P* = .37). Both DASH and SF-36 PCS improved more than the MCID (Figure 3; eTable 10 in the Supplement). Both SHFT and MRC grades correlated positively with SF-36 PCS scores (SHFT: r_s_ = 0.63, MRC: r_s_ = 0.55) and negatively with DASH scores (SHFT: r_s_ = −0.74, MRC: −0.55) (eFigure 5 in the Supplement). After SAN transfers in patients in ICSHT group 0, 2 of 4 patients (50%) completed postoperative DASH and SF-36. Median improvements were 3.3 points in the DASH score and 1.3 points in the SF-36 PCS score. Both did not reach clinical significance (MCID).

Although the MRC grades and SHFT score correlated with the MHQ, SF-36 PCS, and DASH scores at the final follow-up (eFigure 4 and 5 in the Supplement), among the secondary outcome measures, only the DASH score continued to improve beyond 24 months after nerve transfer. The MHQ total score and SF-36 PCS improvement plateaued at 24 months with subtle changes afterward (eTable 10 in the Supplement).

### Adverse Events

One patient had died at the 6-month follow-up of an unrelated event, 1 patient developed syringomyelia that affected recovery from nerve transfer, and 1 patient was lost to follow-up before 24 months. One trapezius donor transiently downgraded to a median MRC of 3 after SAN to triceps nerve transfer and recovered full strength during 12 to 24 months. Two platysma motor branch to triceps, 2 SAN to biceps, and 1 SAN to AIN nerve transfers failed, and patients did not achieve any function, even after long-term follow-up. No other serious adverse events were observed during the study period.

## Discussion

To our knowledge, we present the most comprehensive long-term study of nerve transfers in traumatic cervical SCI to date. First, our results indicate that nerve transfers after SCI facilitate motor recovery and secondarily functional independence. Second, among the nerve transfer options for cervical SCI, supinator to PIN and SAN and axillary nerve to triceps nerve transfers provided consistent motor recovery and relatively faster reinnervation when compared with brachialis to AIN transfers. Third, nerve transfer in chronic SCI is feasible; with careful selection of healthy recipient nerves, good motor outcomes can be achieved, increasing the traditional time window of nerve transfers. Fourth, a continual recovery was observed after nerve transfers for hand reanimation, emphasizing the importance of ongoing nerve regeneration and cortical plasticity and providing critical information for patient counseling.

A lack of rigorous evidence remains for the clinical utility of nerve transfers in tetraplegia. Much of the literature on this topic consists of case reports and retrospective case series limited by small sample sizes.^31,32^ Previous studies used nonstandardized assessments, and substantial heterogeneity in outcomes precluded the quantitative synthesis of existing data.^31,33^ In addition, long-term postoperative data are lacking on nerve transfers in tetraplegia. Importantly, the role of nerve transfers in chronic SCI and high-level cervical SCI remains unknown.^13^

In this study, standardized patient selection and a responsive cohort with long follow-up periods allowed detailed assessment of outcomes after nerve transfers in tetraplegia. At each visit, we observed an objective improvement in hand function, which correlated with improved satisfaction and independence after nerve transfer surgery. Overall, these functional gains exceeded the MCID, suggesting a clinically meaningful improvement after nerve transfers in tetraplegia.

To maximize functional gains, participants underwent double or triple nerve transfers that targeted elbow extension and hand grasp, pinch, and release. Restoration of these functions can provide independence in ADLs, including feeding, self-catheterization, and object manipulation (eg, using the telephone).^5^ Among these nerve transfers, triceps reinnervation resulted in a quicker reinnervation and consistent antigravity function. Achieving antigravity (MRC grade ≥3) strength in elbow extension translates into effective range of motion, which augments reach and allows locking of elbows into extension to facilitate self-transfer capability.^34^ Particularly in high-level injuries (C4 and above, ICSHT 0 group) for which no other surgical options are available, recovery of triceps function can maximize volitional control of the upper limb, which can augment orthosis and other assistive therapies.^35,36^

The hand opening reanimation via supinator to PIN transfer is an important advancement in reconstructive options for tetraplegia. Reanimation of hand opening can augment the grasp function, increasing the range of functional activity.^9^ This nerve transfer resulted in consistently powerful recovery of function and quicker reinnervation. In contrast, brachialis to AIN transfer showed relatively heterogenous outcomes. This nerve transfer enables hand grasp by targeting motor function primarily to the median-innervated flexor digitorum profundus and the flexor pollicis longus.^37^ In some patients, because this is a fascicular-level transfer (Figure 1), there may also be reinnervation to the flexor digitorum superficialis and flexor carpi radialis because the flexor digitorum superficialis/flexor carpi radialis and fascicle reside juxtaposed to the anterior interosseous fascicle in the proximal median nerve. This nerve transfer was less successful and associated with longer time-to-reinnervation periods,^13,37^ likely because of the long regeneration distance to the target myotomes and significant attrition of axons to more proximal sensory and motor targets.^37^ To avoid these issues, distal nerve transfers using the extensor carpi radialis brevis or supinator as donor nerves are preferred alternative options associated with increased success.^13,37^ However, these donors are often not available in high-level injuries (ICSHT groups 1-2).^16^ In our study, the supinator was preferred for PIN reinnervation, and the extensor carpi radialis brevis should be preserved because it provides passive finger flexion secondary to wrist tenodesis, leaving the brachialis as the only donor option.

Our findings challenge the classic notion that delayed nerve transfers are not feasible, because outcomes of nerve transfers performed in chronic SCI (ie, ≥12 months) were comparable to early nerve transfers.^38^ This finding suggests that with careful selection of healthy recipient nerves, motor function can be maximized in both subacute and chronic SCI.^39^ Nerve transfers performed in upper limbs with a lower level of injuries (ICSHT groups 3-4), which often have intact wrist extension and forearm pronation, resulted in superior motor outcomes compared with higher-level injuries (ICSHT groups 0-2). In addition, despite improvement in triceps motor strength after nerve transfers in patients with ICSHT group 0 function, translation to functional improvement was minimal. This finding suggests that although nerve transfers in high cervical SCI are an important advancement, there is a significant need to develop reinnervation strategies that ensure reliable motor recovery.

Nerve transfers have demonstrated significant benefits in tetraplegia; however, the time to gain in motor function may be lengthy, because axons must grow from the site of nerve transfer to the target muscles.^26^ A total of 82% of participants were followed up for at least 24 months, and finger flexor and extensor recipient motor strength continued to improve over time. Although the improvement in motor strength translated to increased functional measures, after a certain follow-up (ie, 24 months), the improvement in functional measures plateaued, with subtle changes afterward. These findings support the known notion that neural regeneration progress is slow and that, even with surgical intervention, continuous hand therapy and attention to general health throughout this period is important.^10,19^

### Limitations

Our study has several limitations. First, our cohort included patients from a single institution treated by a single surgeon, which limits the generalizability of our findings. Second, our participants underwent multiple nerve transfers on both upper limbs, and because of the small sample size, isolating the effect of each nerve transfer was challenging. Third, our study did not compare the outcomes with the control group of patients with tetraplegia who underwent rehabilitation therapy without surgical intervention. Therefore, the isolated contribution of rehabilitation and nerve transfers on the outcomes could not be determined.

## Conclusions

In this case servies, nerve transfers after traumatic cervical SCI were associated with clinically significant improvements in motor strength and increased functional independence. Our findings suggest that nerve transfers can provide reanimation of upper extremities in tetraplegia after both subacute and chronic SCI. Despite these encouraging results, a larger, multicenter randomized clinical trial is indicated to broadly establish the efficacy of this emerging treatment option for patients with tetraplegia.

## Notes

Supplement. **eFigure 1.** Participant Enrollment Flow **eTable 1.** Inclusion and Exclusion Criteria **eTable 2.** ICSHT Groups, SCI Levels, and Goals of Reinnervation **eTable 3.** Preoperative Electrodiagnostic Assessment **eTable 4.** Study Timeline of Interventions and Assessments **eTable 5.** Manual Motor Testing Using Medical Research Council Grade **eTable 6.** Donor Site Motor Strengths Before and After Nerve Transfers **eTable 7.** Primary Outcomes Following Nerve Transfers in Tetraplegia **eFigure 2.** Clinical Images of Patient Hand Function **eTable 8.** Motor Strength Comparison Between Early and Late Follow-up **eFigure 3.** Motor Strength at Intermediate and Long-term Follow-up Visits **eTable 9.** Primary Outcomes Stratified by Time Interval since SCI and ICSHT **eTable 10.** Secondary Outcomes at Postoperative Follow-up Intervals **eFigure 4.** Correlation Between Hand Function and Michigan Hand Outcome Questionnaire **eFigure 5.** Correlation Between Hand Function and Physical Function, Disability, and Pain
eReferences
