# Supplementary material for: Upper Limb Nerve Transfer Surgery in Patients With Tetraplegia
Source: JAMA Netw Open. 2022 Nov 28;5(11):e2243890. doi: 10.1001/jamanetworkopen.2022.43890 (PMC9706368; doi:10.1001/jamanetworkopen.2022.43890)
Supplement: Supplement. — eFigure 1. Participant Enrollment Flow eTable 1. Inclusion and Exclusion Criteria eTable 2. ICSHT Groups, SCI Levels, and Goals of Reinnervation eTable 3. Preoperative Electrodiagnostic Assessment eTable 4. Study Timeline of Interventions and Assessments eTable 5. Manual Motor Testing Using Medical Research Council Grade eTable 6. Donor Site Motor Strengths Before and After Nerve Transfers eTable 7. Primary Outcomes Following Nerve Transfers in Tetraplegia eFigure 2. Clinical Images of Patient Hand Function eTable 8. Motor Strength Comparison Between Early and Late Follow-up eFigure 3. Motor Strength at Intermediate and Long-term Follow-up Visits eTable 9. Primary Outcomes Stratified by Time Interval since SCI and ICSHT eTable 10. Secondary Outcomes at Postoperative Follow-up Intervals eFigure 4. Correlation Between Hand Function and Michigan Hand Outcome Questionnaire eFigure 5. Correlation Between Hand Function and Physical Function, Disability, and Pain eReferences [file jamanetwopen-e2243890-s001.pdf]

Supplemental Online Content

Javeed S, Dibble CF, Greenberg JK, et al. Upper limb nerve transfer surgery in patients with tetraplegia. *JAMA Netw Open*. 2022;5(11):e2243890. doi:10.1001/jamanetworkopen.2022.43890

eFigure 1. Participant Enrollment Flow
eTable 1. Inclusion and Exclusion Criteria
eTable 2. ICSHT Groups, SCI Levels, and Goals of Reinnervation
eTable 3. Preoperative Electrodiagnostic Assessment
eTable 4. Study Timeline of Interventions and Assessments
eTable 5. Manual Motor Testing Using Medical Research Council Grade
eTable 6. Donor Site Motor Strengths Before and After Nerve Transfers
eTable 7. Primary Outcomes Following Nerve Transfers in Tetraplegia
eFigure 2. Clinical Images of Patient Hand Function
eTable 8. Motor Strength Comparison Between Early and Late Follow-up
eFigure 3. Motor Strength at Intermediate and Long-term Follow-up Visits
eTable 9. Primary Outcomes Stratified by Time Interval Since SCI and ICSHT
eTable 10. Secondary Outcomes at Postoperative Follow-up Intervals
eFigure 4. Correlation Between Hand Function and Michigan Hand Outcome Questionnaire
eFigure 5. Correlation Between Hand Function and Physical Function, Disability, and Pain
eReferences

This supplemental material has been provided by the authors to give readers additional information about their work.

© 2022 Javeed S et al. *JAMA Network Open.*

eFigure 1: Participant Enrollment Flow

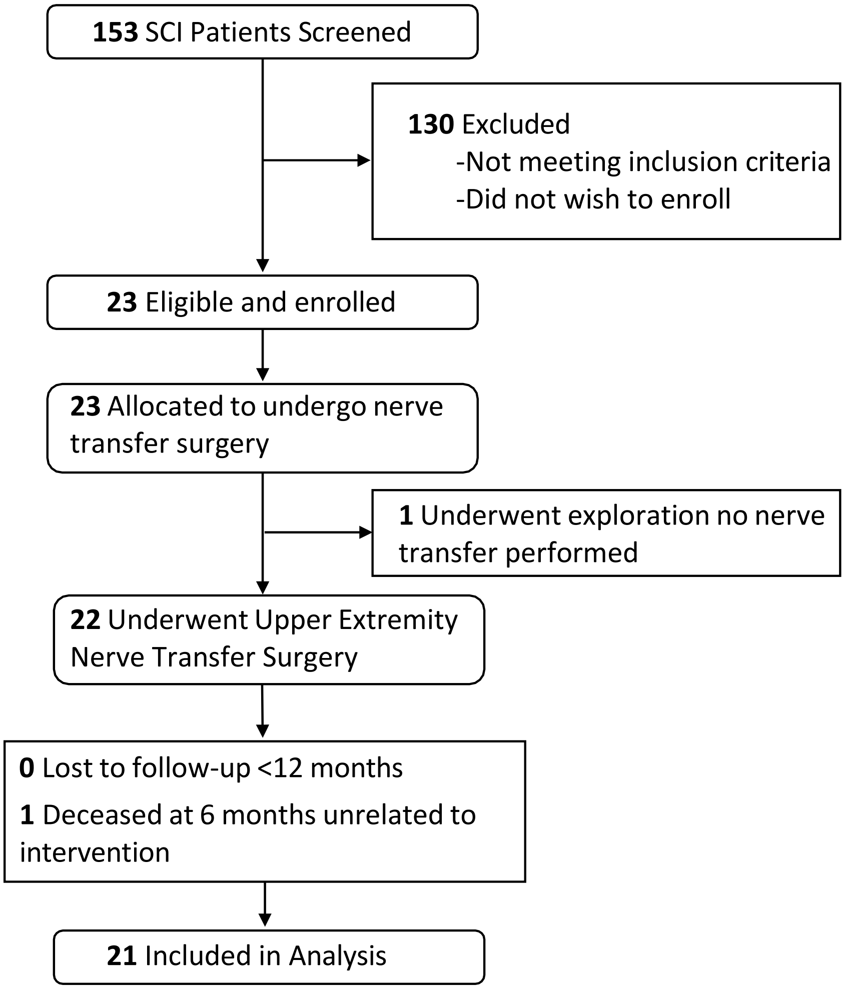

© 2022 Javeed S et al. *JAMA Network Open.*

eTable 1: Inclusion and Exclusion Criteria
Inclusion Criteria<br>1. ≥18 years of age<br>2. Informed Consent Document (ICD) signed by patient<br>3. Cervical spinal cord injury resulting in arm & hand functional impairment, with at least preserved spinal accessory nerve<br>4. Patients with a stable American Spinal Injury Association (ASIA) grade of A, B, or C showing minimal to no evidence of upper extremity functional improvement in motor examination after at least 6 months of non-operative therapy post-injury<br>5. International Classification of Surgery of the Hand in Tetraplegia (ICSHT) category 0 - 4; not applicable for patients with a diagnosis of central cord syndrome<br>6. Willing and able to comply with the study protocol<br>7. At the time of surgery <60 months from spinal cord injury
Exclusion Criteria<br>1. Active infection at the operative site or systemic infection<br>2. Any return or ongoing clinical recovery of distal motor function within 6 months after injury<br>3. Mentally compromised lacking the capacity for treatment decision making<br>4. Currently undergoing long-term steroid therapy<br>5. Significant joint contractures and/or limitations in passive range of motion in the arm or hand<br>6. Active malignancy<br>7. Systemic disease that would affect the patient's welfare or the research study<br>8. Pregnant<br>9. Immunologically suppressed or immunocompromised<br>10. Previous tendon transfers to restore upper extremity function<br>11. Affective disorder of a degree that would make outcome assessment and study participation difficult<br>12. History of brachial plexus injury or systemic neuropathic process

© 2022 Javeed S et al. *JAMA Network Open.*

eTable 2: ICSHT Groups, SCI levels, and Goals of Reinnervation

ASIA	Key Muscles (Grade 3)	ICSHT Group	Key Muscle (Grade 4)
C4 and higher		N/A	
C5	Biceps	0	Biceps
		1	Brachioradialis
C6	Extensor Carpi Radialis Longus (ECRL)	1	Brachioradialis
		2	Extensor Carpi Radialis Longus (ECRL)
		3	Extensor Carpi Radialis Brevis (ECRB)
		4	Pronator Teres (PT)
C7	Triceps	4	Pronator Teres (PT)
		5	Flexor Carpi Radialis (FCR)
		6	Extensor Digitorum Communis (EDC)
		7	Extensor Pollicis Longus (EPL)
C8	Flexor Digitorum Profundus (FDP)	8	Flexor Digitorum Superficialis (FDS)
		9	Flexor Digitorum Superficialis (FDS) and Flexor Digitorum Profundus (FDP)

**ICSHT:0**	Distal spinal accessory nerve to biceps branch of musculocutaneous and axillary branch to the posterior deltoid to triceps branch of the radial nerve – No available tendon transfer option – Goals of reinnervation: elbow flexion and elbow extension.
**ICSHT:1**	Axillary branch to the posterior deltoid to triceps branch of the radial nerve, brachialis branch of the musculocutaneous nerve to anterior interosseous nerve (AIN) fascicle of the median nerve – preserves brachioradialis for future tendon transfer to either augment flexor pollicis longus (FPL) function or transfer to extensor carpi radialis brevis (ECRB) for grasp tenodesis – Goals of reinnervation: elbow extension and key pinch.
**ICSHT:2**	Axillary branch to the posterior deltoid to triceps branch of the radial nerve, brachialis branch of the musculocutaneous nerve to AIN fascicle of the median nerve – preserves brachioradialis for future tendon transfer to either augment flexor pollicis longus (FPL) function or transfer to extensor carpi radialis brevis (ECRB) for grasp tenodesis, extensor carpi radialis longus (ECRL) to flexor digitorum profundus (FDP) for finger flexion – Goals of reinnervation: elbow extension and key pinch.
**ICSHT:3**	Axillary branch of the posterior deltoid to triceps branch of the radial nerve, brachialis branch of the musculocutaneous nerve to AIN fascicle of the median nerve, supinator branches of the radial nerve to posterior interosseous nerve (PIN)– preserves brachioradialis tendon transfer to either augment flexor pollicis longus (FPL) function, extensor carpi radialis longus (ECRL) to flexor digitorum profundus (FDP) for finger flexion – Goals of reinnervation: elbow extension, key pinch, and release.
**ICSHT:4**	Axillary branch of the posterior deltoid to triceps branch of the radial nerve, brachialis branch of the musculocutaneous nerve to AIN fascicle of the median nerve, supinator branches of the radial nerve to posterior interosseous nerve (PIN)– preserves brachioradialis tendon transfer to either augment flexor pollicis longus (FPL) function, extensor carpi radialis longus (ECRL) to flexor digitorum profundus (FDP) for finger flexion, pronator teres (PT) to extensor digitorum communis – Goals of reinnervation: elbow extension and key pinch.

eTable 3: Preoperative Electrodiagnostic Assessment			
**Nerve transfers**	**Donors**	**Recipients**	
	EMG[c]	NCS[a]	EMG[c]
Brachialis to AIN	Biceps/Brachialis EMG	Median nerve CMAP[b] (distal wrist to APB)	AIN innervated flexor EMG[d]
Supinator to PIN	Supinator EMG	Radial nerve CMAP (radial forearm to EIP)	PIN innervated extensor EMG[e]
Axillary/SA to Triceps	Deltoid EMG (Trapezius EMG not done)	Not feasible/tested	Triceps EMG

[a] NCS: Nerve conduction studies
[b] CMAP amplitude in mV
Median CMAP- Normal > 4 mV, Reduced <4 mV, Absent 0 mV
Radial CMAP- Normal > 2 mV, Reduced <2 mV, Absent 0 mV

[c] EMG: electromyography
Spontaneous activity (fibrillations/positive sharp waves/fasciculations),
Volitional Motor Unit Potential morphology (duration/amplitude/polyphasia),
Recruitment pattern characteristics (normal/mildly reduced/moderate-severely reduced/none).
[d] FPL/APB EMG: surrogate for C8/T1 innervation in AIN
[e] EDC/EIP EMG: surrogate for C7/C8 innervation in PIN

Abbreviations: APB: abductor pollicis brevis; AIN: anterior interosseus nerve; CMAP: compound muscle action potential; EDC: extensor digitorum communis; EIP: extensor indicis proprius; EMG: electromyography; FPL: flexor pollicis longus; PIN: posterior interosseus nerve; SA: spinal accessory.

© 2022 Javeed S et al. *JAMA Network Open.*

eTable 4: Study Timeline of Interventions and Assessments									
	**Assessor**	**Baseline**	**6 weeks**	**6 months**	**12 months**	**18 months**	**24 months**	**36 months**	**48 months**
**Screening**									
Inclusion/Exclusion Criteria	Peripheral Nerve Surgeon	X							
International Standards for Neurological Classification of Spinal Cord Injury (ISNCSCI)	Senior Physiatrist	X							
International Classification for Surgery of the Hand in Tetraplegia (ICSHT)	Peripheral Nerve Surgeon	X							
**Preoperative**									
Electrodiagnosis[a]	Senior Electrophysiologist	X							
Functional Electrical Stimulation[a]	Peripheral Nerve Surgeon	X							
Intraoperative Neuromuscular Stimulation[a]	Peripheral Nerve Surgeon	X							
**Research Visits**									
Motor Strength– Manual Muscle Testing (MMT)	Hand Surgeon and Occupational Therapist	X	X	X	X	X	X	X	X
Range of motion[a]	Hand Surgeon and Occupational Therapist	X	X	X	X	X	X	X	X
Sollerman Hand Function Test (SHFT)	Occupational Therapist	X	X	X	X	X	X	X	X
Michigan Hand Outcome Questionnaire (MHQ)	Patient Reported	X	X	X	X	X	X	X	X
Disabilities of Arm, Shoulder, and Hand (DASH)	Patient Reported	X	X	X	X	X	X	X	X
Short Form–36 (SF36)	Patient Reported	X	X	X	X	X	X	X	X
Serious Adverse Events	Research Coordinator		X	X	X	X	X	X	X

[a] Data are not reported in this manuscript

© 2022 Javeed S et al. *JAMA Network Open.*

eTable 5: Manual Motor Testing using Medical Research Council Grade	
**Description**	**Grade**
No contraction observed or palpated	0/5
Muscle twitch observed or palpated	1/5
Full movement, gravity eliminated and no resistance	2/5
Full movement against gravity, no resistance	3/5
Moderate resistance against gravity throughout the range of movement.	4/5
Maximal resistance against gravity throughout the range of movement.	5/5

© 2022 Javeed S et al. *JAMA Network Open.*

eTable 6: Donor Site Motor Strengths before and after Nerve Transfers						
**Nerve Transfers**	**Donor Sites**	**Pre-operative**	**2 Months**	**12 Months**	**24 months**	**p value[a]**
Brachialis to AIN	Elbow Flexion (n=25)	5 (5-5)	5 (4-5)	5 (5-5)	5 (5-5)	0.2
Supinator to PIN	Supination (n=19)	5 (5-5)	5 (4-5)	5 (4.3-5)	5 (4.2-5)	0.8
Axillary nerve to Triceps	Shoulder Abduction (n=4)	5 (4-5)	5 (4-5)	5 (4-5)	5 (4-5)	0.2
SA nerve to Triceps/Biceps	Shoulder Shrug (n=5)	5 (4-5)	3 (3-5)	3 (3-3.5)	4 (3-4.5)	0.2

Motor Strength in medical research council grades. Data reported as Median (IQR).
[a] Preoperative *vs.* 12 months postoperative measures analyzed via paired Wilcoxon signed rank test.

Abbreviations: AIN: Anterior interosseus nerve; PIN: Posterior interosseus nerve; SA: Spinal Accessory.

© 2022 Javeed S et al. *JAMA Network Open.*

eTable 7: Primary Outcomes Following Nerve Transfers in Tetraplegia (n=21 patients)									
	**Preoperative Strength**				**Postoperative Strength**				
**Nerve Transfer**	**Muscle**	**MRC**	**N (%)**		**Muscle**	**MRC**	**N (%)**		**p value[a]**
**Brachialis to AIN**	**FDP**	0	24 (96)	0 (0-0)[b]	**FDP**	0	1 (4)	2 (1-3)	<0.001
		1	1 (4)			1	6 (24)		
		2	0 (0)			2	8 (32)		
		3	0 (0)			3	6 (24)		
		4	0 (0)			4	4 (16)		
		5	0 (0)			5	0 (0)		
	**FPL**	0	24 (96)	0 (0-0)	**FPL**	0	1 (4)	2 (1-3)	<0.001
		1	1 (4)			1	10 (40)		
		2	0 (0)			2	5 (20)		
		3	0 (0)			3	4 (16)		
		4	0 (0)			4	5 (20)		
		5	0 (0)			5	0 (0)		
	**FCR**	0	14 (56)	0 (0-1)	**FCR**	0	10 (40)	1 (0-2)	0.015
		1	8 (32)			1	3 (12)		
		2	1 (4)			2	6 (24)		
		3	0 (0)			3	1 (4)		
		4	2 (8)			4	2 (8)		
		5	0 (0)			5	3 (12)		
**Supinator to PIN**	**EDC**	0	19 (100)	0 (0-0)	**EDC**	0	1 (5)	4 (2-4)	<0.001
		1	0 (0)			1	4 (21)		
		2	0 (0)			2	2 (11)		
		3	0 (0)			3	1 (5)		
		4	0 (0)			4	9 (47)		
		5	0 (0)			5	2 (11)		
	**EPL/B**	0	17 (89)	0 (0-0)	**EPL/B**	0	1 (5)	4 (3-4)	<0.001
		1	2 (11)			1	2 (11)		
		2	0 (0)			2	2 (11)		
		3	0 (0)			3	4 (21)		
		4	0 (0)			4	9 (47)		
		5	0 (0)			5	1 (5)		
	[c]**ECRB**	0	1 (5)	4 (2-5)	[c]**ECRB**	0	1 (5)	4.5 (4-5)	0.16
		1	1 (5)			1	1 (5)		
		2	3 (16)			2	0 (0)		
		3	5 (26)			3	0 (0)		
		4	2 (11)			4	7 (37)		
		5	7 (37)			5	10 (53)		
**SA/Ax to Triceps**	**Triceps**	0	8 (80)	0 (0-0.2)	**Triceps**	0	2 (20)	3 (2.5-4)	0.011
		1	1 (10)			1	0 (0)		
		2	1 (10)			2	1 (10)		
		3	0 (0)			3	4 (40)		
		4	0 (0)			4	2 (20)		
		5	0 (0)			5	1 (10)		

[a] Paired Wilcoxon Signed Rank tests were used to detect the significance of change preoperative versus postoperative motor strengths.
[b] Median (IQR).
[c] ECRB is not the direct recipient and downgrade in function is a concern secondary to Supinator-PIN transfer.

Abbreviations: AIN: Anterior interosseus nerve; Ax: Axillary nerve; EDC: extensor digitorum communis; EPL/B: Extensor pollicis longus/brevis; ECRB: Extensor carpi radialis brevis; FCR: Flexor carpi radialis; FDP: Flexor digitorum profundus; FPL: Flexor pollicis longus; MRC: Medical research council grades; PIN: Posterior interosseus nerve; SA: Spinal Accessory nerve.

© 2022 Javeed S et al. *JAMA Network Open.*

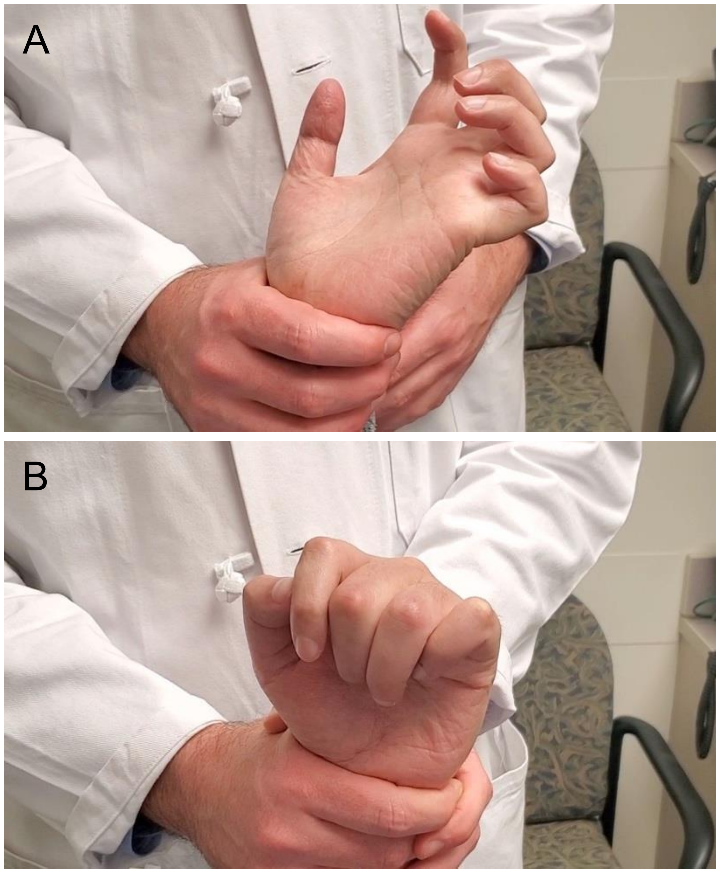

eFigure 2: Full range of hand function after 24 months of follow-up. Patient underwent double nerve transfer to reanimate hand opening by supinator nerve branch to posterior interosseus nerve transfer and brachialis motor branch to anterior interosseus nerve transfer. **A)** Hand opening, **B)** Grasp and hand closing.

Participant 16 (27 years old male, neurological level C4, ASIA A, ICSHT group 3) nerve transfer surgery performed 10 months following spinal cord injury.

© 2022 Javeed S et al. *JAMA Network Open.*

eTable 8: Motor Strength Comparison between Early and Late Follow-up							
**Muscles**	**N**	**12 months**	**N**	**24 months**	**N**	**48 months**	**p value**[a]
**Brachialis to AIN**							
Flexor digitorum profundus	25	1 (0-1)	25	2 (1-2)	13	3 (2-3)	<0.001
Flexor pollicis longus	25	1 (0-1)	25	1 (1-2)	13	3 (1-4)	<0.001
**Supinator to PIN**							
Extensor digitorum communis	19	2 (0-3)	19	3.5 (2-4)	10	4 (3-4)	0.003
Extensor pollicis longus/brevis	19	2 (1-3)	19	3.5 (3-4)	10	4 (3-4)	0.004
**SA/Ax to Triceps**							
Triceps	10	3 (1-3)	10	3 (2-4)	5	3 (0-4)	0.15

Motor Strength in medical research council grades. Data are median (IQR)
[a] Friedman test was performed to assess the trend in improvement across multiple assessments.

Abbreviations: AIN: Anterior interosseus nerve; Ax: Axillary nerve; PIN: Posterior interosseus nerve; SA: Spinal Accessory nerve.

© 2022 Javeed S et al. *JAMA Network Open.*

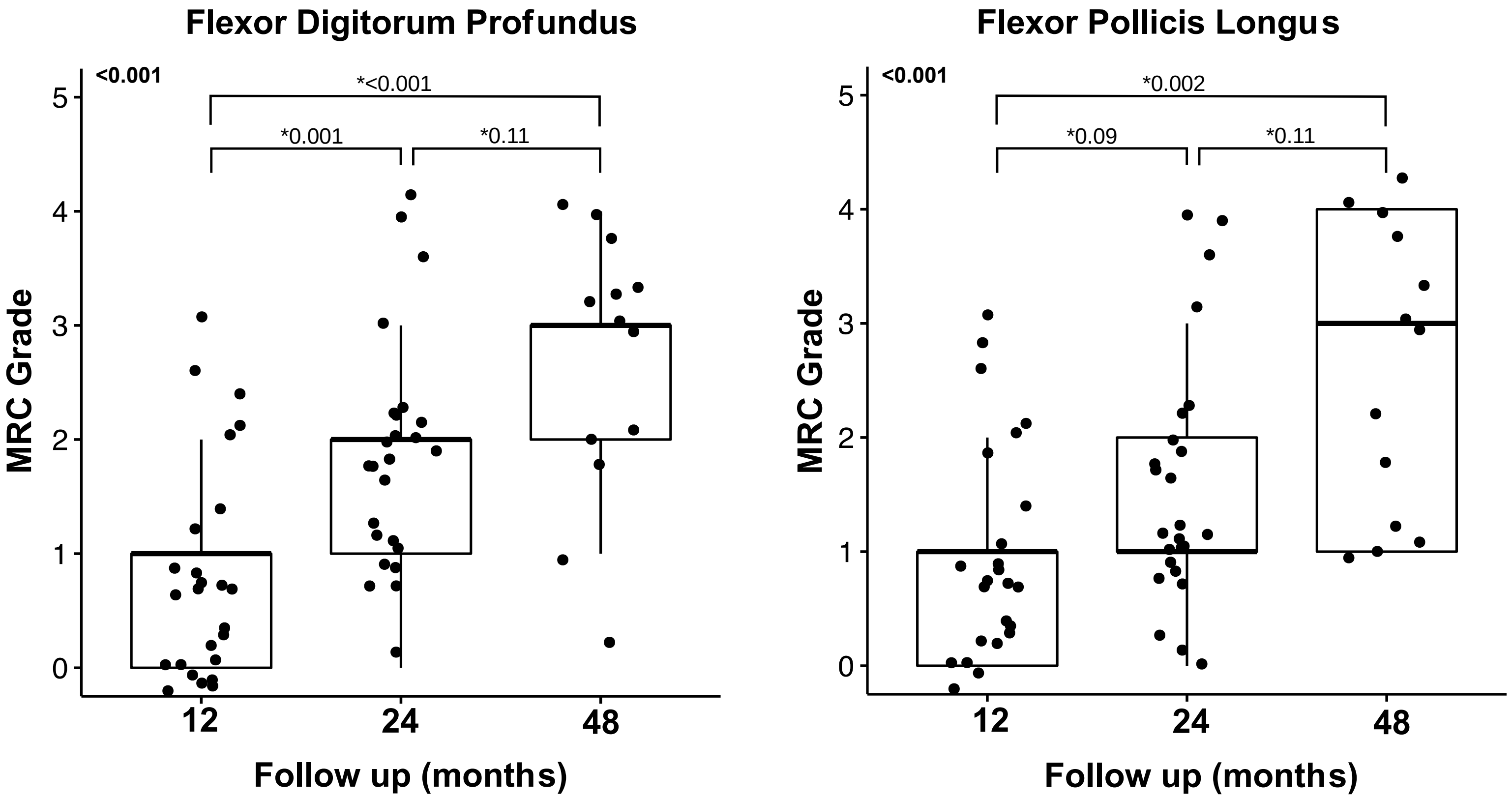

eFigure 3A: Motor Strength medical research council (MRC) grades following Brachialis to AIN nerve transfer at intermediate and long-term follow-up visits. Each dot represents a recipient muscle tested with motor strength in MRC grades 0-5. To assess the trend in improvement of motor strength over repeated measures at 12-, 24-, and 48-months, the Friedman test was used (P values in bold). Pairwise comparisons between 12–48, 12–24, and 24–48-month time points were evaluated by post-hoc analysis using pairwise Wilcoxon rank sum tests with Bonferroni correction (p values in asterisk).

© 2022 Javeed S et al. *JAMA Network Open.*

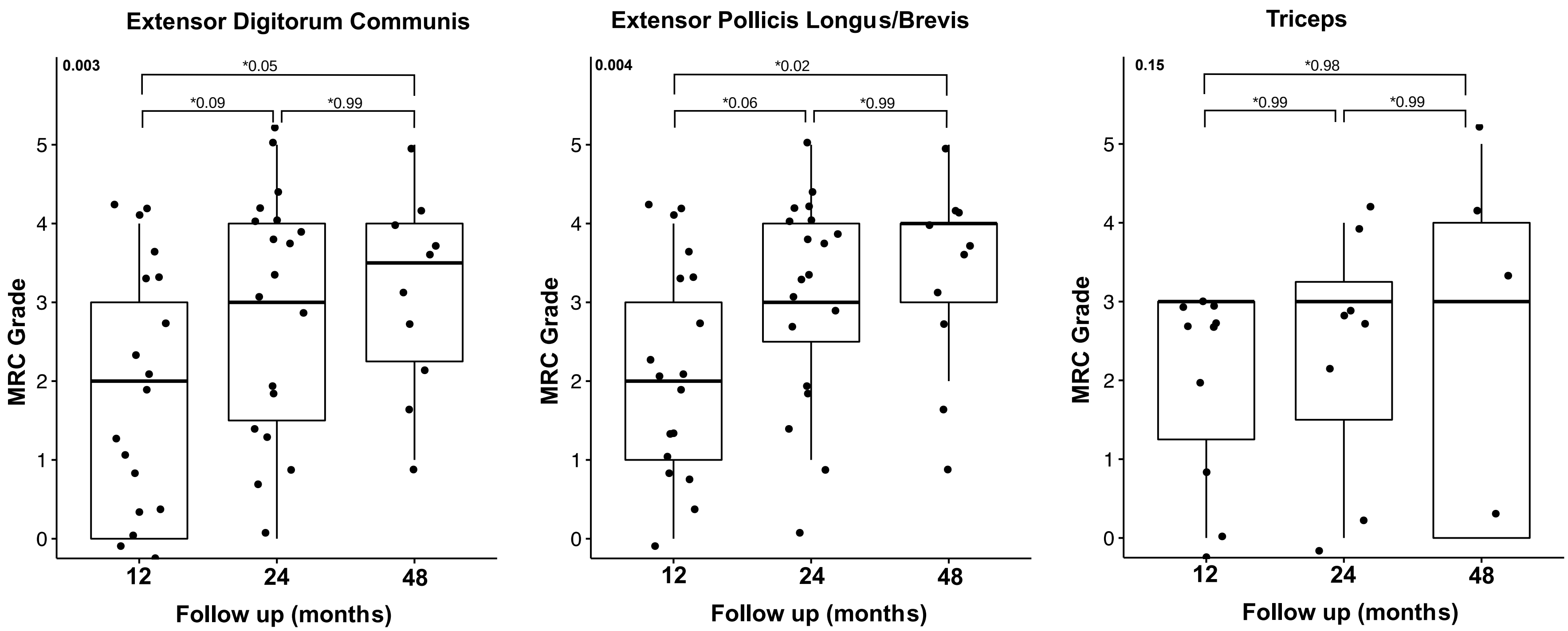

eFigure 3B: Motor Strength medical research council (MRC) grades following Supinator to PIN and Triceps nerve transfer at intermediate and long-term follow-up visits. Each dot represents a recipient muscle tested with motor strength in MRC grades 0-5. To assess the trend in improvement of motor strength over repeated measures at 12-, 24-, and 48-months, the Friedman test was used (P values in bold). Pairwise comparisons between 12–48, 12–24, and 24–48-month time points were evaluated by post-hoc analysis using pairwise Wilcoxon rank sum tests with Bonferroni correction (p values in asterisk).

© 2022 Javeed S et al. *JAMA Network Open.*

eTable 9: Primary Outcomes Stratified by Time Interval since SCI and ICSHT

Stratified by time interval since SCI					
	**number of transfers**	**within 12 months**	**number of transfers**	**more than 12 months**	**p value[a]**
		**Median MRC**		**Median MRC**	
All nerve transfers	16	2 (0-4)	42	3 (2-4)	0.25
Nerve transfers for grasp and pinch	8	3 (2-4)	17	4 (2-3.2)	-
Nerve transfers for hand opening[b]	4	4 (3-4)	15	3 (3-4)	-
Nerve transfers for elbow extension[b]	3[c]	0 (0-2)	7	3 (3-5)	-
**Stratified by ICSHT**					
	**number of transfers**	**High level[d] (0-2)**	**number of transfers**	**Low level[d] (3-4)**	**p value[a]**
All nerve transfers	23	2 (1-3)	35	4 (3-4)	<0.001
Nerve transfers for grasp and pinch	8	2 (1-2)	17	4 (2-4)	-
Nerve transfers for hand opening[b]	5	3 (1-4)	14	4 (3-4)	-
Nerve transfers for elbow extension[b]	7	3 (1-3.5)	3	3 (3-3.5)	-

Motor Strength in medical research council grades. Data are median (IQR)
[a] Mann-Whitney U test. Statistical test was not done in nerve transfer sub-groups due to small sample size.
[b] Only best motor strength achieved was included among the muscles innervated by recipient nerves AIN (FDP and FPL) and PIN (EDC and EPL/B).
[c] Two nerve transfers were platysma motor branch to triceps motor branch of radial nerve.
[d] International classification of the surgery of hand in tetraplegia (ICSHT) was classified as high-level injury with groups 0-2 and low-level injury with groups 3-4. ICSHT group classification in eTable 2.

Abbreviations: AIN: Anterior interosseus nerve; EDC: extensor digitorum communis; EPL/B: Extensor pollicis longus/brevis; FDP: Flexor digitorum profundus; FPL: Flexor pollicis longus; MRC: Medical research council grades; PIN: Posterior interosseus nerve.

© 2022 Javeed S et al. *JAMA Network Open.*

eTable 10: Secondary Outcomes at Postoperative Follow-up Intervals								
**Assessment Tool**		**Baseline**	**12 months**	**24 months**	**48 months**	**Final**	**Δ Improvement**	**p value[b]**
**Hand Function**								
**Solleman Hand Function Test[a]**		20 (0-31)	25 (5-35)	29.5 (8.75-40.5)	45 (12-45)	27 (9-37)	5[c] (2–9.5)	0.011
Number of upper limbs		15	15	10	3	15	15	
**Michigan Hand Outcome Questionnaire**	Total MHQ Score	32.2 (23.4-43.2)	42 (25-49)	42.5 (29.6-54.2)	45.6 (39.2-68)	39.8 (32.5-64.7)	15[d] (8–22)	<0.001
	Hand Function	15 (5-30)	22.5 (5-46.2)	35 (3.7-46.2)	42.5 (20-58.7)	25 (0-47.5)	10 (5–20)	<0.001
	Activities of Daily Living	0 (0-5)	0 (0-16.2)	20 (0-35)	15 (0-50)	10 (0-35)	15 (5–22.5)	0.004
	Work Performance	0 (0-20)	0 (0-20)	20 (0-58.7)	30 (0-63.7)	15 (0-55)	17.5 (0–37.5)	0.012
	Aesthetics	62.5 (50-93.7)	56.2 (43.7-84.4)	68.7 (50-75)	68.7 (54.7-100)	68.7 (53.1-90.6)	9.4 (0–22)	0.06
	Satisfaction with hand function	16.7 (8.3-33.3)	35.4 (16.6-62.5)	29.2 (8.3-54.2)	50 (46-66.7)	45.8 (16.6-62.5)	21 (12.5–29.2)	<0.001
	Pain	0 (0-40)	5 (0-21.25)	0 (0-30)	0 (0-15)	0 (0-35)	-2.5 (15–0)	0.35
	Number of upper limbs	29	22	23	16	29	29	-
**Disability and Quality of Life**								
**Disabilities of Arm, Hand, and Shoulder**		84.6 (75.6-87.7)	71.7 (64.2-81.7)	74.7 (63.3-79.2)	60.4 (41.7-82.5)	69.2 (57.5-84.2)	13.3[e] (7.9–21.7)	<0.001
Number of Patients		19	16	16	10	19	19	
**Short Form 36v2 Summary Scores**	Physical Component Summary	27.1 (24.4-33.1)	30.9 (26.8-34.4)	31.1 (27.7-37.2)	29.5 (23.8-38.4)	33.0 (27.3-38.3)	5.6[f] (1.3–8.2)	0.03
	Mental Component Summary	58.6 (46.9-63.9)	57 (50.4-61.3)	57.1 (45.1-65.6)	53.4 (40.1-64.6)	57.3 (49.4-63.9)	2.1 (-3.3–6.6)	0.37
Number of Patients		19	16	16	10	19	19	-

Data reported as Median (IQR). Time points of follow-up in months. Δ Improvement is reported as Median difference (95% Confidence Intervals)
[a] SHFT was completed by only participants who underwent nerve transfers to reanimate hand function due to its relevancy to measure the hand function specifically.
[b] Preoperative *vs.* final postoperative scores were analyzed via Wilcoxon Singed Rank Tests.
[c] Minimal Clinically Important Difference (MCID) of SHFT score[1]: 4.9 points. MCID of SHFT score defined by distribution-based method SEM= SD * sqrt(1-R), where SD is standard deviation of baseline SHFT score, and R is the test-retest reliability of SHFT. R for SHFT is established to be 0.98.[1] A conservative R of 0.9 was used
[d] Minimal Clinically important difference (MCID) of MHQ Score[2]: 9.3 points, Hand function: 7.7 points, ADL: 14.7 points, Work performance: 13.7 points, Satisfaction with hand function: 20 points, and Pain: 15.7 points
[e] MCID of DASH score[3]: 10.83 points
[f] MCID of SF36 PCS and MCS[4]: 4 points

© 2022 Javeed S et al. *JAMA Network Open.*

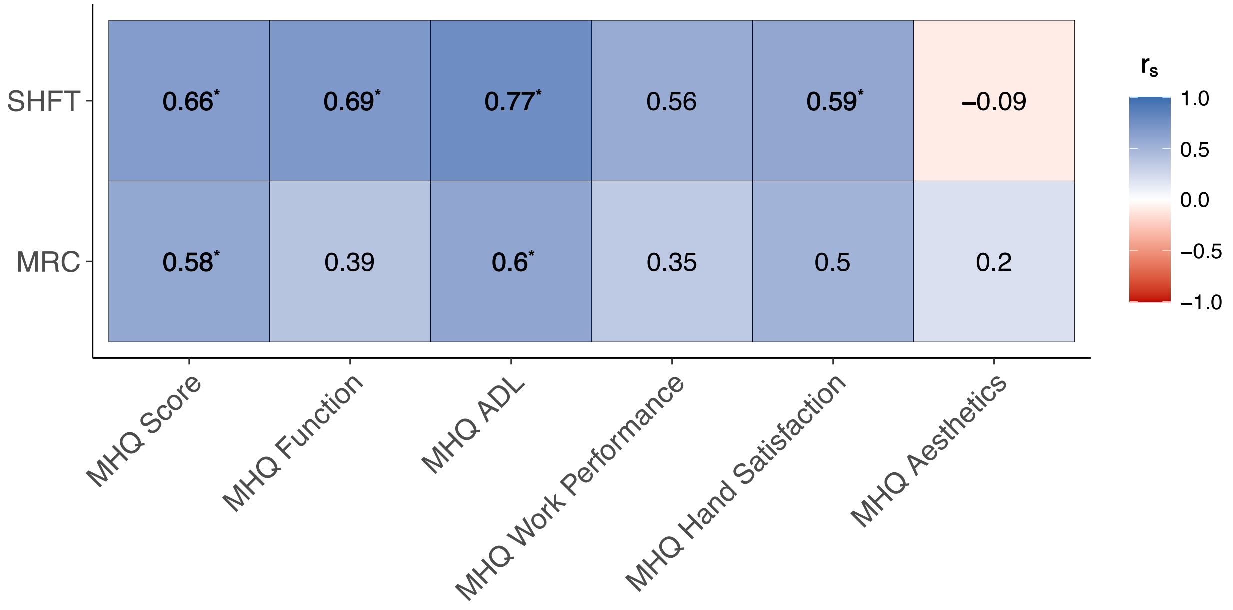

eFigure 4: Correlation matrix showing the association of Sollerman hand function test and motor strength (MRC) with patient reported Michigan hand outcome questionnaire (MHQ) subcategories. Only best motor strengths were included among the nerve transfers performed on each limb (n=29). Gradient bar represents the strength of spearman rho's correlation coefficient (r_s) from negative (red) to positive (blue). P values were adjusted by Bonferroni correction for multiple comparisons with adjusted alpha *p<0.001. Significant correlations are bold with asterisk.

ADL: activities of daily living; MHQ: Michigan hand outcome questionnaire; MRC: Medical research council grade; SHFT: Sollerman hand function test.

© 2022 Javeed S et al. *JAMA Network Open.*

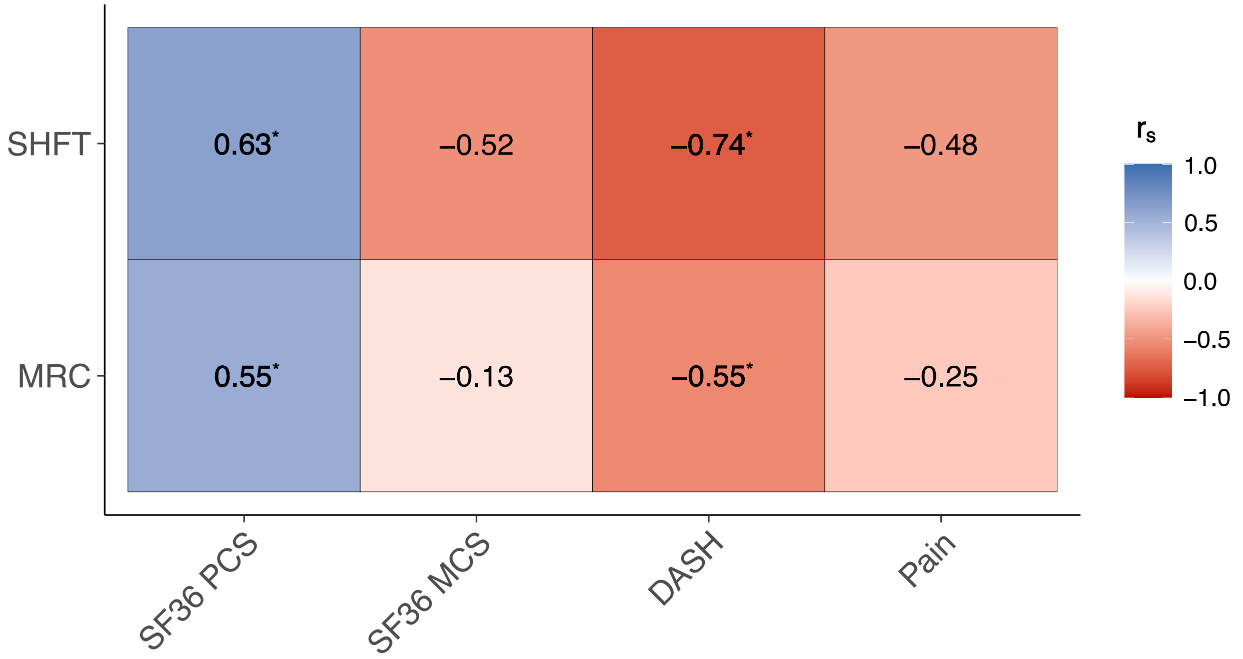

eFigure 5: Correlation matrix showing the association of Sollerman hand function test and motor strength (MRC) with SF36 physical component summary, mental component summary, disability rating by DASH, and pain. Only best motor strengths were included among the nerve transfers performed on each limb (n=29). Gradient bar represents the strength of spearman rho's correlation coefficient (r_s) from negative (red) to positive (blue). P values were adjusted by Bonferroni correction for multiple comparisons with adjusted alpha *p<0.003. Significant correlations are bold with asterisk.

DASH: disability of arm, shoulder; MCS: Mental component summary; MRC: Medical research council grade; PCS: physical component summary; SHFT: Sollerman hand function test.

© 2022 Javeed S et al. *JAMA Network Open.*

eReferences

1. Sollerman C, Ejeskär A. Sollerman hand function test. A standardised method and its use in tetraplegic patients. *Scand J Plast Reconstr Surg Hand Surg*. Jun 1995;29(2):167-76. doi:10.3109/02844319509034334
2. Koopman JE, van Kooij YE, Selles RW, et al. Determining the Minimally Important Change of the Michigan Hand outcomes Questionnaire in patients undergoing trigger finger release. *J Hand Ther*. Jul 24 2021;doi:10.1016/j.jht.2021.06.003
3. Franchignoni F, Vercelli S, Giordano A, Sartorio F, Bravini E, Ferriero G. Minimal clinically important difference of the disabilities of the arm, shoulder and hand outcome measure (DASH) and its shortened version (QuickDASH). *J Orthop Sports Phys Ther*. Jan 2014;44(1):30-9. doi:10.2519/jospt.2014.4893
4. Badhiwala JH, Witiw CD, Nassiri F, et al. Minimum Clinically Important Difference in SF-36 Scores for Use in Degenerative Cervical Myelopathy. *Spine (Phila Pa 1976)*. Nov 1 2018;43(21):E1260-e1266. doi:10.1097/brs.0000000000002684

© 2022 Javeed S et al. *JAMA Network Open.*
